# Assessing the effectiveness of advanced platelet rich fibrin in treating gingival recession: a systematic review and meta-analysis

**DOI:** 10.1186/s12903-024-05115-7

**Published:** 2024-11-19

**Authors:** Wafaa Saleh, Marwa Abdelhaleem, Samah Elmeadawy

**Affiliations:** 1 Oral Medicine, Periodontology, Diagnosis and Oral Radiology Department, Faculty of Dentistry, Mansoura University, Mansoura, 33516 Egypt; 2 Oral Medicine, Periodontology, Diagnosis and Oral Radiology Department, Faculty of Dentistry, Horus University, Horus, Egypt

**Keywords:** Advanced platelet rich fibrin, Gingival recession, Treatment, Systematic review

## Abstract

**Objectives:**

The literature lacks comprehensive evidence on the efficacy of advanced platelet rich fibrin(A-PRF) in treating gingival recession. Therefore, this systematic review and meta-analysis aimed to evaluate the effectiveness of A-PRF in the treatment of gingival recession.

**Materials and methods:**

We adhered to the guidelines of PRISMA in searching the following databases: PubMed/MEDLINE, Embase, Cochrane Library, Web of Science, and Scopus to include all the eligible studies according to the prespecified inclusion and exclusion criteria. We conducted our search up to February 28, 2024. We conducted a meta-analysis of the primary and secondary clinical outcomes to measure the changes from baseline to 6 months after surgery.

**Results:**

Our review included 10 randomized clinical trials in which 146 participants with 457 recession defects were included. We found that combination of A-PRF with various surgical techniques, such as coronally advanced flap (CAF) connective tissue graft (CTG), VISTA, tunneling, and pinhole surgical technique, demonstrated promising outcomes but varied by comparison group. We observed that CTG with CAF showed a higher reduction in recession depth in comparison to A-PRF with CAF. This review indicated no statistical or clinical differences in recession width, width of keratinized gingiva, probing depth, and clinical attachment level between the study and control groups.

**Conclusions:**

Due to the less invasive nature of A-PRF, it provides a better clinical option to improve the outcomes of treating gingival recession. However, more well-designed RCTs with standardized approaches are needed to confirm these results.

## Introduction

Gingival recession is defined as the unesthetic exposure of the root surface due to the downward displacement of the gingival margin as a result of various etiological factors. Among these etiologic factors, microbial influence on periodontal tissues, traumatic teeth brushing, mispositioned teeth, and improper tooth movement during orthodontic treatment have been reported as the most common causes of gingival recession [1–4]. Replacement of the lost gingival tissue was performed through various surgical and non-surgical approaches [5–7]. 

The gold standard treatment modalities of gingival recession are free gingival grafts and connective tissue grafts (CTGs). Minimally invasive techniques along with pedicle flaps and tunneling strategies have come to be brought within the discipline of periodontology in recent years due to their high patient acceptance and decrease tissue morbidity. In addition, current regenerative treatment approaches, along with enamel matrix derivatives and guided tissue regeneration have been brought aiming to stimulate tissue growth and restore the natural structure of the gingiva [8–10]. 

Platelet concentrates have gained widespread utilization due to their capacity to release bioactive molecules, cytokines, and proteins upon activation, thereby facilitating the healing process. Autologous platelet concentrates (APCs) are obtained through the centrifugation of venous blood at varying speeds, with the addition of thrombin and anticoagulants. The primary types of APCs include platelet-rich plasma (PRP), platelet-rich fibrin (PRF), and concentrated growth factor (CGF) [11–13]. 

Recent advances in fundamental research on PRF have elevated to include new modifications and editions, including Advanced PRF (A-PRF), Leukocyte PRF (L-PRF), Injectable PRF (i-PRF), and Titanium-PRF (T-PRF). These variants differ primarily in their centrifugation speeds, time, and tube substances, leading to more valuable tissue regeneration. For instance, A-PRF is produced using slower centrifugation, resulting in more concentration of leukocytes and increase growth factors, which improve wound healing and tissue regeneration. I-PRF offers a greater fluid consistency, making it simpler to mix with biomaterials or inject into treatment sites. L-PRF consists of a dense but porous fibrin network, containing leukocytes and aggregates of activated platelets that are distributed throughout the clot. T-PRF, organized in titanium tubes, yields a better release of growth factors with superior bone and tissue regeneration. Combining PRF variations with regenerative materials, along with bone grafts or stem cells enhances the clinical outcomes particularly bone and tissue regeneration [13–19]. 

A-PRF is a new modification of PRF that has been developed due to the limitations detected in the conventional PRF [11, 20]. Researchers have begun to compare special adjustments of PRF with a purpose to decide which formulations offer the high-quality regenerative consequences [14, 21]. The introduction of low-speed centrifugation has facilitated the production of A-PRF characterized by a porous fibrin structure with a higher concentration and sustained release of growth factors and cytokines than conventional PRF. Moreover, it has a uniform distribution in terms of platelet and leukocyte quantity, as well as the dispersal of these cells across the entire membrane than PRF. Therefore, it has been broadly applied in soft and hard tissue surgeries. The fibrin matrix offers a scaffold for cellular migration, proliferation, and tissue regeneration [13, 15–17, 22, 23]. 

A-PRF is characterized by its capability to improve wound healing and tissue regeneration. Unlike earlier forms of PRF, A-PRF uses a lower centrifugation speed and longer time, these criteria enhance the tissue regeneration and improve the treatment outcomes [14, 21]. 

In the context of gingival recession therapy, the regenerative capacity of A-PRF is applied to stimulate the growth of gingival tissues, promoting more proper wound healing and the restoration of a healthful periodontium [14, 17, 21, 24]. Various studies has shown the effectiveness of A-PRF in the treatment of gingival recession with improvement of the clinical outcomes and enhancement of the patient’s esthetics and satisfaction [14, 24–26]. 

While several systematic reviews have already been published that examine the impact of PRF in gingival recession treatment [27–30], to our information, none have included the different variants of PRF nor in particular centered on A-PRF. This more recent variation of PRF presents specific biological properties, and our goal turned to isolate and study its specific impact on gingival recession without confounding results from different PRF variants. We didn’t Include different forms of PRF to focus our review and analyze the precise consequences of A-PRF, which is a new modification that has not been systematically evaluated in the field of gingival recession treatment.

Despite being a new modification of the well-known platelet concentrates that gained a growing popularity in treatment of gingival recession, the available literature lacks a comprehensive evaluation of the effectiveness of A-PRF in improving the clinical outcomes of gingival recession treatment. Hence, we intended to fill this gap by carefully evaluating all the available clinical studies. We believe that we are the first to do a comprehensive and methodical search of relevant literature in order to evaluate the research that has looked at the application of A-PRF in gingival recession therapies.

## Methods

### Protocol registration

The present systematic review followed the guidelines defined in the Preferred Reporting Items for Systematic Reviews and Meta-Analyses (PRISMA). This review’s protocol, meticulously describing its methodology, has been officially registered with PROSPERO (International Prospective Register of Systematic Reviews) identification code CRD42024523720.

### Study PICOS questions

The formulated PICOS (Population, Intervention, Comparison, Outcomes, and Study design) question can be expressed as follows:

#### Population(P)

The population of interest includes individuals diagnosed with gingival recession. This includes participants of any age, gender, or ethnicity.

#### Intervention(I)

The intervention includes treatment of gingival recession with A-PRF.

#### Comparison(C)

Comparison interventions include conventional treatments such CTGs, collagen membrane, L-PRF, Pinhole Surgical technique as well as Coronally advanced flap (CAF) alone. These interventions are compared with the use of A-PRF for the treatment of gingival recession.

#### Outcomes(O)

The review assesses various clinical parameters related to the efficacy of A-PRF, including gingival recession depth (RD), recession width (RW), Width of keratinized gingiva (WKG), Probing depth (PD) and Clinical attachment level (CAL).

#### Study design(S)

The systematic review includes randomized controlled trials (RCTs), with follow-ups scheduled at 3 months, and/or 6 months.

### Identification of databases and search strategy

Two individuals performed the electronic search in the following database such as Embase, Cochrane Library, PubMed/MEDLINE, Web of Science, and Scopus. We conducted our search up to February 2024 using the following search terms: (“advanced PRF” OR “A-PRF”) AND (“gingival recession” OR " gingival recession treatment " OR “gingival recession therapy” OR " root coverage “). In addition, we searched the latest edition of the well-known journals in the field of periodontology. Moreover, we didn’t restrict our search by date or language.

### Inclusion and exclusion criteria

#### Inclusion criteria

Studies were included if they met the following criteria:

1. Studies involving treatment of gingival recession in human participants.
2. Patients’ age is more than 18 years old.
3. studies used A-PRF for the treatment of gingival recession.
4. Studies in which A-PRF was compared with conventional therapies or placebo.
5. Studies involving participants with no systemic diseases and currently on medications that might affect the outcome of gingival recession treatment.

#### Exclusion criteria

1. In vitro studies, Animal studies and reviews articles.
2. Studies focus only on other forms of platelet-rich fibrin or platelet rich plasma.
3. Studies with insufficient data on A-PRF application technique or outcomes.
4. Case reports, case series, and retrospective studies.
5. Studies with a follow-up period of less than 3 months.
6. Publications not available in English or with no access to translation services.

### Articles selection process

Two independent reviewers (WS&MA) chose the qualified articles by reading the title and the abstracts. The potentially eligible articles underwent reading of the full text to extract the probable eligible studies based on the prespecified inclusion and exclusion criteria. We consulted the third reviewer (SE) if we found a disagreement between the first two reviewers, then we held a comprehensive discussion to include the articles.

### Data extraction

Data collection was performed using an Excel spreadsheet (Microsoft Excel, version 2010) to collect detailed information from the included studies for qualitative synthesis. Data extraction was independently conducted by two reviewers (WS & MA) and further revised by third reviewer (SE). We extracted study characteristics such as design and sample size, participant demographics including recession type and location, intervention details such as surgical techniques and use of adjunctive treatments. Tables 1 and 2.

The outcomes assessed included RD, RW, WKG, PD, and CAL at various time points, and results including percentage of root coverage (%RC) as well as authors’ conclusions regarding treatment efficacy. Table 2.

**Table 1:** Study design of included studies

Author and Year	Study Design	Surgical technique		Centrifugation Speed/Time
		Study group	Control group	
Jain et al. 2021	Randomized clinical trial	VISTA + A-PRF	VISTA + Collagen membrane	1500 rpm for 14 min
Tadepalli et al. 2022	Randomized clinical trial	CAF + A-PRF	CAF + L-PRF	1500 rpm for 14 min
Anegundi et al. 2023	Randomized clinical trial	Tunnel technique + A-PRF	Tunnel technique + CTG	1300 rpm for 8 min
Trivedi et al. 2023	Randomized clinical trial	Pinhole surgical technique + A-PRF	Pinhole surgical technique	1500 rpm for 14 min
Öngöz Dede et al. 2023	Randomized clinical trial	CAF + A-PRF	CAF alone	1500 rpm for 14 min
Jagtap et al. 2023	Randomized clinical trial	CAF + A-PRF	CAF alone	1500 rpm for 14 min
Silva et al. 2023	Randomized clinical trial	Tunnel technique + A-PRF	Tunnel technique + CTG	1500 rpm for 14 min
Abu-Ta’a 2023	Randomized clinical Trial	CAF + A-PRF	CAF + CTG	1300 rpm for 14 min
Durgapal & Shetty 2023	Randomized clinical Trial	VISTA + A-PRF	VISTA + Collagen membrane	1500 rpm for 12 min
Nadal et al. 2024	Randomized clinical Trial	CAF + A-PRF	CAF + CTG	1500 rpm for 14 min

VISTA: Vestibular incision subperiosteal tunnel Access

CAF: Coronally advanced flap

CTG: Connective tissue graft

Rpm: Revolutions per minute

L-PRF: Leukocyte-Platelet Rich Fibrin

**Table 2:** General characteristics of included studies

Author & Year	Recession Type	Recession location	Number of Patients	Smokers	Number of Recession defects (study & Control)	Age range in years	Male(M)/ Female(F)	Follow-up (Months)	% of Root Coverage (%RC) at the end of study		Authors conclusion	
									Study group	Control group		
Jain et al. 2021	Miller’s class I & II	NA	8	Excluded	20	18–50	N/A	3&6 Months	77.5 ± 46.5	61.67± 25.20	VISTA with both A-PRF and collagen membrane showed good clinical outcomes, but better results were obtained with A-PRF.	
Tadepalli et al. 2022	Miller’s class I & II	Maxillary anterior teeth and premolars	30	Excluded	30	18–65	18 M 12 F	3&6 Months	81.66 ± 28.21	67.20 ± 32.81	No statistically significant differences in therapeutic outcomes between the two groups (CAF + A-PRF and CAF + L-PRF)	
Anegundi et al. 2023	Cairo class 1 (RT1)	N/A	17	Excluded	34	38.06 ± 4.47	10 M 7 F	3&6 Months	51.96 ± 15.45	84.31 ± 17.89	Both CTG and A-PRF can be used in treating gingival recessions. CTG is a better material in achieving root coverage and increasing KTW.	
Trivedi et al.2023	Miller’s class I & II	Bilateral on the upper arch	25	Excluded	165	18–55	15 M 10 F	6&12 Months	86.31 ± 26.02	72.31 ± 38.25	The test group showed a better resolution of gingival recession in comparison to control group.	
Öngöz Dede et al. 2023	Cairo class 1(RT1)	Maxillary anterior teeth and premolars	11	Excluded	30	25–45	5 M 5 F	6 Months	80%	53.33%	A-PRF is superior to CAF alone in terms of outcomes of gingival recession treatment.	
Jagtap et al. 2023	Miller’s class-II	N/A	20	Excluded	20	N/A	N/A	1 & 3 Months	98.8 ± 3.79	95.9 ± 8.71	The additional use of A-PRF membrane did not provide additional benefits in terms of root coverage outcomes compared with CAF alone.	
Silva et al. 2023	Cairo class 1(RT1)	NA	NA	Excluded	44	23–47	NA	3&6 Months	NA	NA	Both groups showed improvement in periodontal measures, yet there was no significant statistical difference between them.	
Abu-Ta’a 2023	Miller’s class I&II	Canine & premolars	15	Excluded	30	N/A	12 M 3 F	3 & 6 Months	69.2 ± 22.9	88.66 ± 33.18	This study demonstrates that A-PRF and CTG effectively manage gingival recession defects. However, CTG resulted in better clinical outcomes in terms of reduction in recession height and width.	
Durgapal& Shetty 2023	Cairo class 1 & 2	Maxillary anterior teeth and premolars	10	NA	44	18–50	NA	6 Months	86.09 ± 17.11	76.95 ± 17.11	The VISTA along with A-PRF and collagen membrane showed improvement of the clinical outcomes with more promising results observed in the A-PRF group	
Nadal et al. 2024	Cairo class 1	Anterior and posterior teeth	10	Excluded	40	23–47	2 M 8 F	6 Months	54.3%	73%	Although there wasn’t a statistically significant gap, the control group showed slightly higher rates of gingival gain in both height and thickness	

### Data synthesis and meta-analysis

We performed both qualitative and quantitative analysis on the collected data. Tables 1 and 2 present demographic information and qualitative data from all included studies. When at least two trials had comparable outcome measurements and follow-up times, quantitative data for various outcomes were gathered and underwent a meta-analysis. Table 3.

Review Manager 5.3 (RevMan 5.3, Version 5.3.5, Copenhagen, Denmark: The Nordic Cochrane Centre, The Cochrane Collaboration, 2014) was used to examine the quantitative data.

The selection of statistical model was determined by the level of heterogeneity between studies. The fixed-effect model was used while research was considered homogeneous, assuming that any variant between them was because of random chance. This became implemented while the I² statistic was underneath 50% and the P-value passed 0.05, indicating low heterogeneity. Conversely, the random-effect model was implemented while there has been tremendous heterogeneity (I² > 50% and *P* < 0.05).

**Table 3:** Clinical characteristics of included studies

Author and Year	Groups	Recession depth (RD) (mm)		Recession width (RW)(mm)		Width of keratinized gingiva (WKG) (mm)		Probing depth (PD)(mm)		Clinical attachment level (CAL) (mm)	
		Baseline	6 months	Baseline	6 months	Baseline	6 months	Baseline	6 months	Baseline	6months
Jain et al. 2021	Study group	2.05 ± 0.83	0.35 ± 0.53	N/A		4.40 ± 1.17	5.00 ± 1.55	0.90 ± 0.32	0.70 ± 0.26	5.45 ± 1.74	4.50 ± 1.27
	Control group	2.00 ± 0.33	0.80 ± 0.59			4.30 ± 0.95	5.15 ± 1.06	0.90 ± 0.32	0.50 ± 0.00	5.55 ± 2.35	5.20 ± 2.45
Tadepalli et al. 2022	Study group	2.63 ± 0.82	0.53 ± 0.915	3.93 ± 0.70	1.27 ± 1.87	1.47 ± 0.74	2.13 ± 0.743	1.27 ± 0.45	1.07 ± 0.258	3.93 ± 0.96	1.47 ± 1.187
	Control group	2.53 ± 0.74	0.87 ± 0.834	3.43 ± 0.63	1.40 ± 1.24	1.60 ± 0.73	2.60 ± 0.507	1.60 ± 0.63	1.07 ± 0.258	4.13 ± 1.12	1.93 ± 0.961
Anegundi et al. 2023	Study group	2.35 ± 0.49	1.12 ± 0.49	2.94 ± 0.43	1.35 ± 0.70	3.12 ± 0.70	3.88 ± 0.93	1.53 ± 0.51	1.24 ± 0.44	3.88 ± 0.60	2.35 ± 0.60
	Control group	3.06 ± 0.56	0.53 ± 0.62	3.24 ± 0.83	0.59 ± 0.71	2.82 ± 0.72	4.29 ± 0.47	1.47 ± 0.51	0.88 ± 0.33	4.53 ± 0.62	1.29 ± 0.85
Trivedi et al. 2023	Study group	1.49 ± 0.66	0.42 ± 0.56	2.51 ± 0.68	0.99 ± 0.87	3.08 ± 0.72	4.20 ± 0.75	0.97 ± 0.52	0.64 ± 0.46	2.46 ± 0.80	1.06 ± 0.76
	Control group	1.36 ± 0.57	0.51 ± 0.62	2.47 ± 0.58	1.07 ± 0.77	3.06 ± 0.56	3.87 ± 0.69	0.90 ± 0.36	0.54 ± 0.49	2.25 ± 0.69	1.05 ± 0.81
Öngöz Dede et al. 2023	Study group	2.43 ± 1.04	0.26 ± 0.59	4.73 ± 1.53	0.66 ± 1.49	3.46 ± 0.91	4.80 ± 1.37	1.56 ± 0.49	1.80 ± 0.41	4.00 ± 1.14	2.06 ± 0.70
	Control group	2.26 ± 1.16	0.46 ± 0.74	3.80 ± 1.01	1.26 ± 1.94	3.06 ± 1.22	4.73 ± 0.79	1.66 ± 0.48	1.86 ± 0.51	4.13 ± 1.27	2.33 ± 1.04
Jagtap, et al. 2023	Study group	3.18 ± 1.36	NA	N/A		1.43 ± 0.62	NA	N/A		N/A	
	Control group	2.39 ± 0.81				2.08 ± 0.57					
Silva et al. 2023	Study group	2.13 ± 0.83	1.13 ± 0.51	2.68 ± 0.64	1.53 ± 0.63	1.37 ± 0.47	2.33 ± 0.81	1.71 ± 0.38	1.08 ± 0.26	3.18 ± 0.85	2.13 ± 0.51
	Control group	2.04 ± 0.57	0.93 ± 0.45	2.5 ± 0.51	1.26 ± 0.59	1.36 ± 0.49	2.2 ± 0.67	0.71 ± 0.52	1.06 ± 0.13	3.18 ± 0.73	2.13 ± 0.74
Abu-Ta’a 2023	Study group	2.53 ± 0.74	0.87 ± 0.83	3.40 ± 0.63	1.40 ± 1.24	2.60 ± 0.73	2.53 ± 0.64	1.10 ± 0.63	1.20 ± 0.25	4.13 ± 1.12	1.33 ± 0.96
	Control group	2.87 ± 0.83	0.27 ± 0.70	3.87 ± 0.51	0.53 ± 1.4	2.40 ± 0.50	3.20 ± 0.77	1.07 ± 0.25	1.17 ± 0.25	3.93 ± 0.96	1.93 ± 0.72
Durgapal& Shetty 2023	Study group	2.59 ± 0.5	0.36 ± 0.49	NA		4.5 ± 0.51	6.73 ± 0.46	1.91 ± 0.68	1.5 ± 0.51	4.59 ± 0.91	1.73 ± 0.7
	Control group	2.59 ± 0.51	0.68 ± 0.5			4.55 ± 0.51	6.55 ± 0.51	1.82 ± 0.66	1.41 ± 0.5	4.41 ± 0.91	1.64 ± 0.58
Nadal et al. 2024	Study group	2.15 ± 1.03	1.00 ± 0.91	3.15 ± 1.42	2.00 ± 1.74	NA		1.35 ± 0.53	1.36 ± 0.60	3.20 ± 1.28	2.15 ± 1.03
	Control group	2.30 ± 0.86	0.70 ± 0.80	3.00 ± 1.45	1.70 ± 1.94			1.40 ± 0.58	1.40 ± 0.64	3.45 ± 0.94	2.00 ± 1.07

**Fig. 1:**
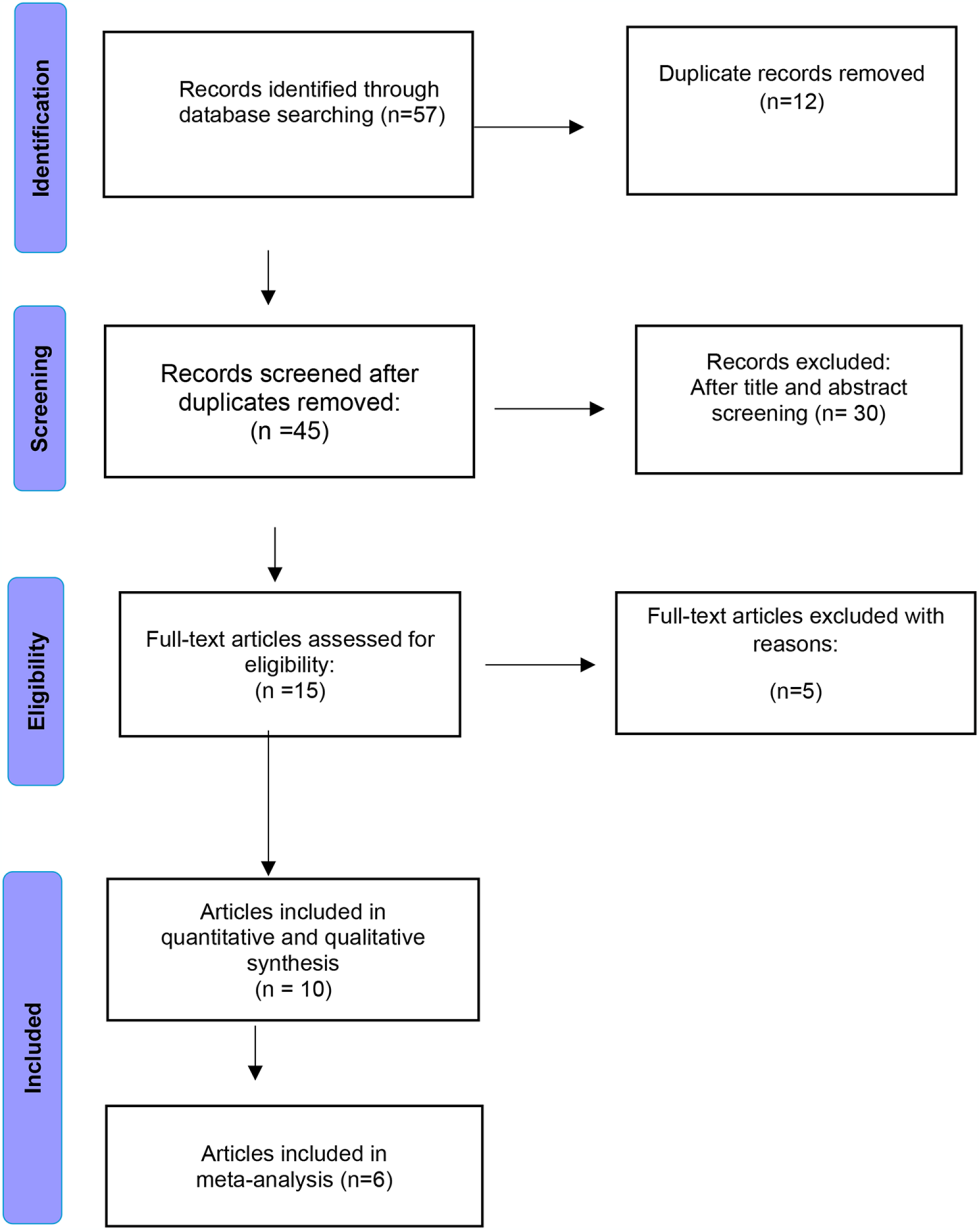
PRISMA flow diagram of the included studies

**Fig. 2:**
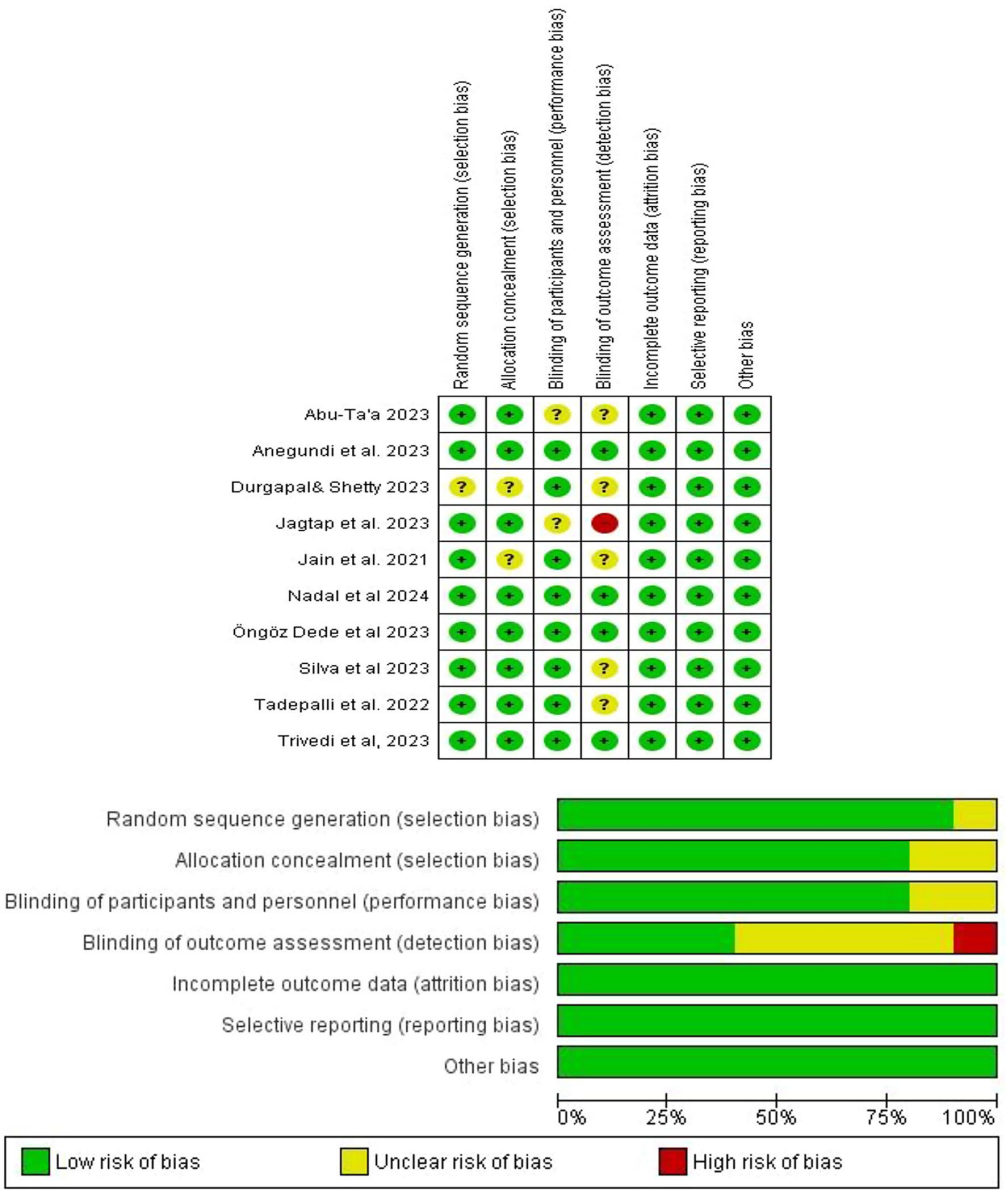
Risk of bias graph

## Results

In our systematic review and meta-analysis, we identified a total of 57 articles. After removing duplicate records (*n* = 12), 45 records underwent screening. Among these, 30 records were excluded as they did not meet the inclusion criteria. Following the title and abstract screening, 15 full-text articles were assessed for eligibility. Of these, 5 articles were excluded, and the reasons for exclusion were documented. Consequently, 10 studies met the inclusion criteria and were included in qualitative analysis. Of these 10 articles, 6 were included in the quantitative (meta-analysis synthesis) as illustrated in Fig. 1: Study flowchart.

### Design of the included studies

Ten RCTs including 146 patients with age range18-65 years we included in the current review. We found 457 recession defects in the included studies. Among the included articles, five studies evaluated the effectiveness of A-PRF in combination with CAF, four studies utilized A-PRF with tunneling or VISTA techniques, and one combined A-PRF with the pinhole surgical technique. Several surgical techniques were included in the control groups including CTG, L-PRF, CAF alone, tunneling technique, and collagen membrane.

### Type of interventions

We found a variety of surgical techniques and comparisons between different intervention groups (Table 1). Jain et al. 2021 and Durgapal & Shetty,2023 assessed the efficacy of VISTA combined with A-PRF compared to VISTA with a collagen membrane [31, 32]. Anegundi et al. 2023 and Silva et al.2023 compared the tunnel technique combined with A-PRF to the same technique combined with CTG [33, 34]. Trivedi et al.2023 evaluated the effectiveness of pinhole surgical technique with A-PRF against pinhole surgical technique alone [25]. Jagtap et al. 2023 and Öngöz Dede et al. 2023 studied the effects of CAF combined with A-PRF versus CAF alone [24, 26]. Abu-Ta’a 2023 and Nadal et al. 2024 studied CAF with A-PRF versus CAF combined with CTG [35, 36]. Finally, Tadepalli et al. 2022 studied the effect of CAF + A-PRF versus CAF + L-PRF [17]. 

### Location of the gingival recession

The location of gingival recession differed across the 10 RCTs. Four studies did not detail the location of gingival recession(N/A) [26, 31, 33, 34]. Three studies treated the gingival recession in the maxillary anterior teeth and premolars only [17, 24, 32]. One study targeted bilateral recession on the upper arch [25]. Furthermore, one study treated gingival recession in the canine and premolar areas [35]. Lastly, one study studied recession in anterior and posterior teeth [36]. 

### Follow up

Different follow-up duration was reported among the 10 included RCTs. some studies conducted a follow-up at 3 months and 6-months or 6 and 12 months after treatment. Other studies performed designed the follow-up at 1 and 3 months or at 6 months only.

### Preparation of A-PRF

Preparation of A-PRF was performed using centrifugation durations and speeds that showed slight variations among the 10 RCTs. The most used preparation protocol is 1500 rpm for 14 min [17, 24–26, 31, 34, 36]. One study followed a protocol 1300 rpm for 14 min [35], while one study used 1300 rpm for 8 min [33]. Moreover, one study used 1500 rpm for 12 min [32]. 

### Risk of bias assessment

The included studies were assessed for risk of bias by two independent reviewers (WS and MA) initially, and then further reviewed by a third reviewer (SE) using the Cochrane tool [37]. RCTs were categorized as low risk of bias if all domains were at low risk, unclear risk of bias if one or fewer domains were at unclear risk, and high risk of bias if one or more domains were at high risk. The risk of bias graph is shown in Fig. 2.

### Meta-analysis of the primary and secondary outcomes after 6 months follow-up

From the 10 RCTs, 6 studies with similar comparison of the surgical techniques and similar outcomes were included in the meta-analysis. We divided the outcomes of the gingival recession treatment into primary and secondary outcomes. The primary outcomes included gingival RD, RW, WKG, and secondary outcomes included PPD and CAL. For the primary and secondary outcomes, mean differences (MD) and standard deviations (SD) were computed. The average difference between the baseline and 6-month follow-up of the treatment sites reported in each study was used to characterize changes in all outcomes. The contribution of each study was weighted appropriately, and the fixed effect model or random effects model was selected based on the apparent heterogeneity amongst studies. To summarize group differences, forest plots were created, with a significant threshold of *P* < 0.05. In accordance with the Cochrane Handbook for Systematic Reviews, heterogeneity was evaluated using the 𝜒2 test and the I^2^ statistics test.

### Meta-analysis of the primary outcomes: RD, RW, and KTW

All outcomes were evaluated at a 6-month follow-up compared to baseline across the included studies.

#### Recession depth

**Fig. 3:**
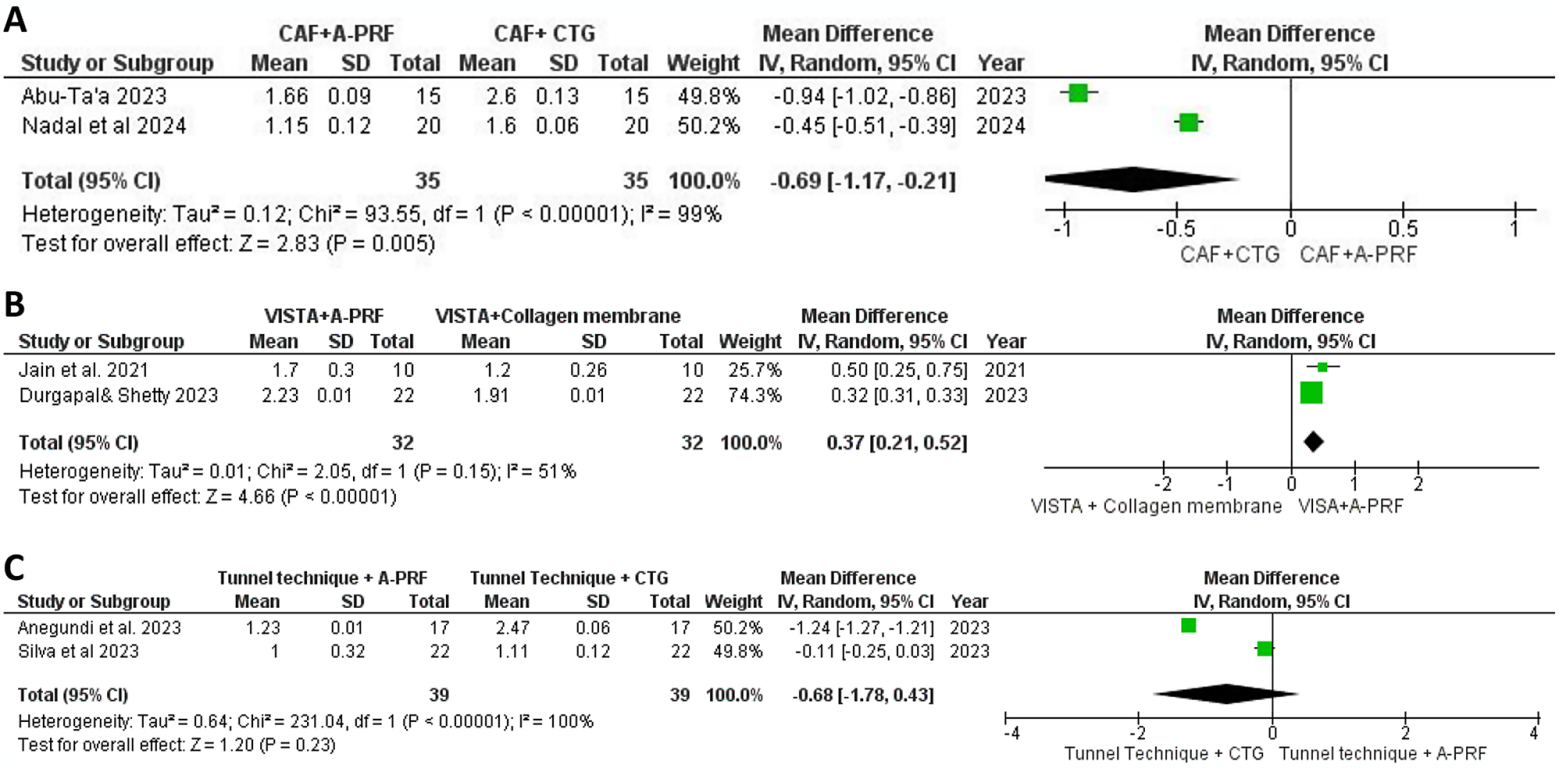
Forest plot for the RD reduction after 6 months. **A**: Comparison between CAF + A-PRF vs. CAF + CTG. **B**: Comparison between VISTA + A-PRF vs. VISTA + collagen membrane. **C**: Comparison between tunnel technique + A-PRF vs. tunnel technique + CTG

At the 6-month follow-up, our meta-analysis of RD outcomes from studies comparing CAF with A-PRF to CAF with CTG (*n* = 35 per group) [35, 36] revealed a MD of0.69 mm (95% CI: -1.17, -0.21) using a random-effects model to address heterogeneity (I² = 99%). A statistically significant difference favored the CAF and CTG group (*p* = 0.005; Fig. 3A). In two RCTs comparing VISTA with A-PRF to VISTA with a collagen membrane [31, 32], the meta-analysis showed a significant RD difference favoring VISTA and A-PRF, with an MD of 0.37 mm (95% CI: 0.21, 0.52) and heterogeneity (I² = 51%; Fig. 3B). Similarly, our analysis of two RCTs comparing the tunnel technique with A-PRF versus the tunnel technique with CTG found no statistically significant difference in RD [33, 34], with an MD of 0.68 mm (95% CI: -1.78, 0.43) and substantial heterogeneity (I² = 100%; Fig. 3C).

#### Recession width

**Fig. 4:**
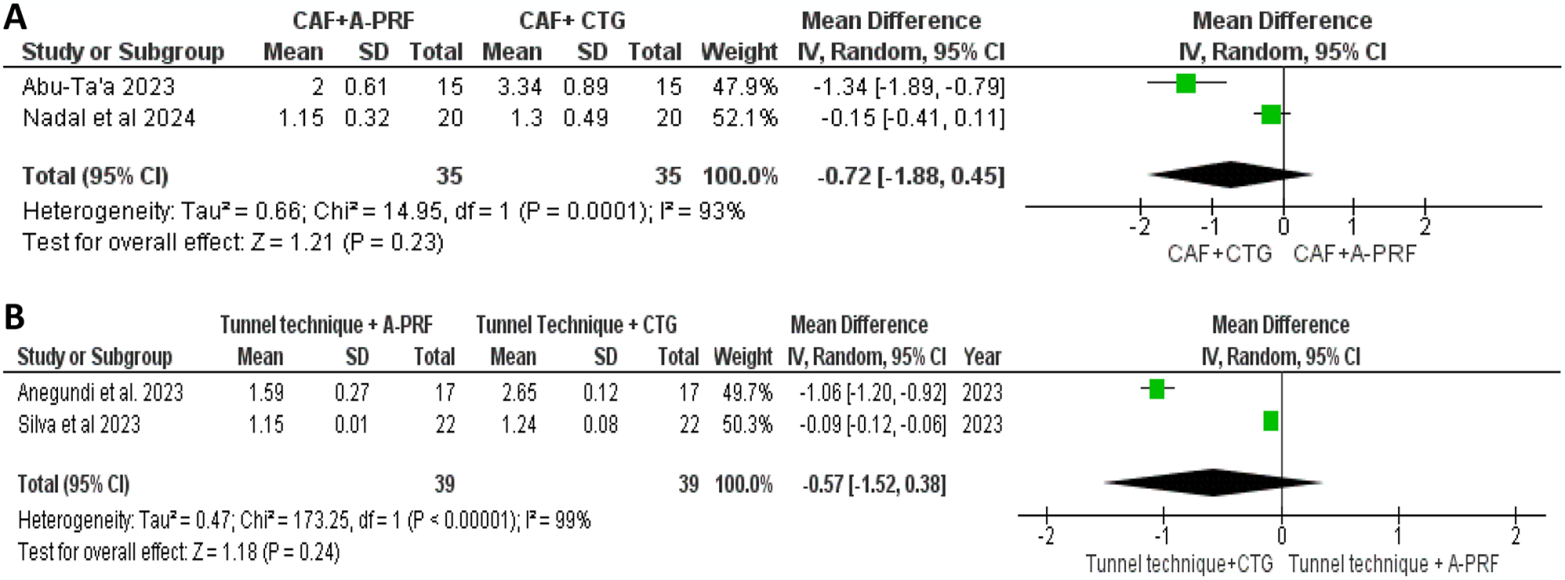
Forest plot for the RW change after 6 months. **A**: Comparison between CAF + A-PRF vs. CAF + CTG. **B**: Comparison between tunnel technique + A-PRF vs. tunnel technique + CTG

Figure 4A illustrates the forest plot of meta-analysis results of RW at 6 months in which the CAF with A-PRF was compared to CAF with CTG. The MD in RW between the groups was 0.72 (95% CI: -1.88, 0.45), with no significant difference between the study and control groups. We found a Significant heterogeneity (I² = 93%), and the random-effects model showed no statistically significant effect (*P* = 0.23) [35, 36]. In Fig. 4B, the meta-analysis comparing the tunnel technique with A-PRF to the tunnel technique with CTG showed an MD of 0.57 (95% CI: -1.52, 0.38), with no significant difference. Although the detected high heterogeneity (I² = 99%), the random-effects model revealed no significant effect (*P* = 0.24) [33, 34]. 

#### Width of keratinized gingiva

**Fig. 5:**
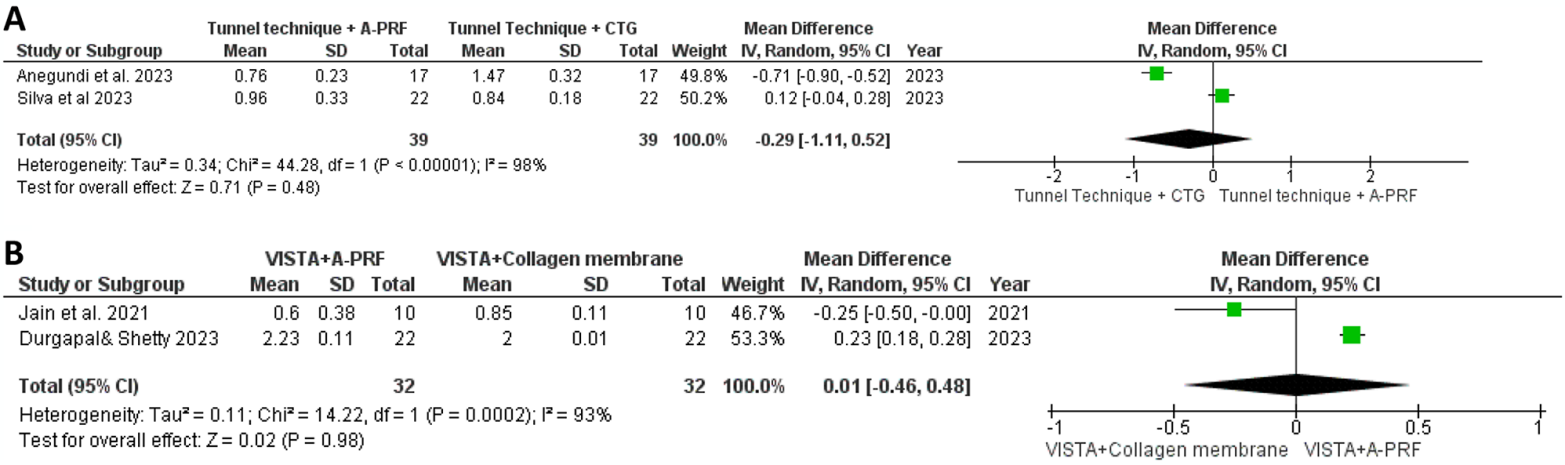
Forest plot for the WKG changes after 6 months. **A**: Comparison between tunnel technique + A-PRF vs. tunnel technique + CTG. **B**: Comparison between VISTA + A-PRF vs. VISTA + collagen membrane

In Fig. 5A, the meta-analysis comparing WKG at 6 months between the tunnel technique with A-PRF and the tunnel technique with CTG showed a mean difference of 0.29 (95% CI: -1.11, 0.52). The analysis revealed no statistically significant difference(*P* = 0.48), with substantial heterogeneity observed (I² = 98%) [33, 34]. Similarly, Fig. 5B presents the meta-analysis comparing VISTA with A-PRF to VISTA with a collagen membrane on WKG at 6 months, revealing a mean difference of 0.01 (95% CI: -0.46, 0.48). The analysis found no significant difference between the study and control groups (*P* = 0.98), despite considerable heterogeneity (I² = 93%), suggesting variability in study outcomes [31, 32]. 

### Meta-analysis of the secondary outcomes: PPD, CAL

#### Periodontal probing depth

**Fig. 6:**
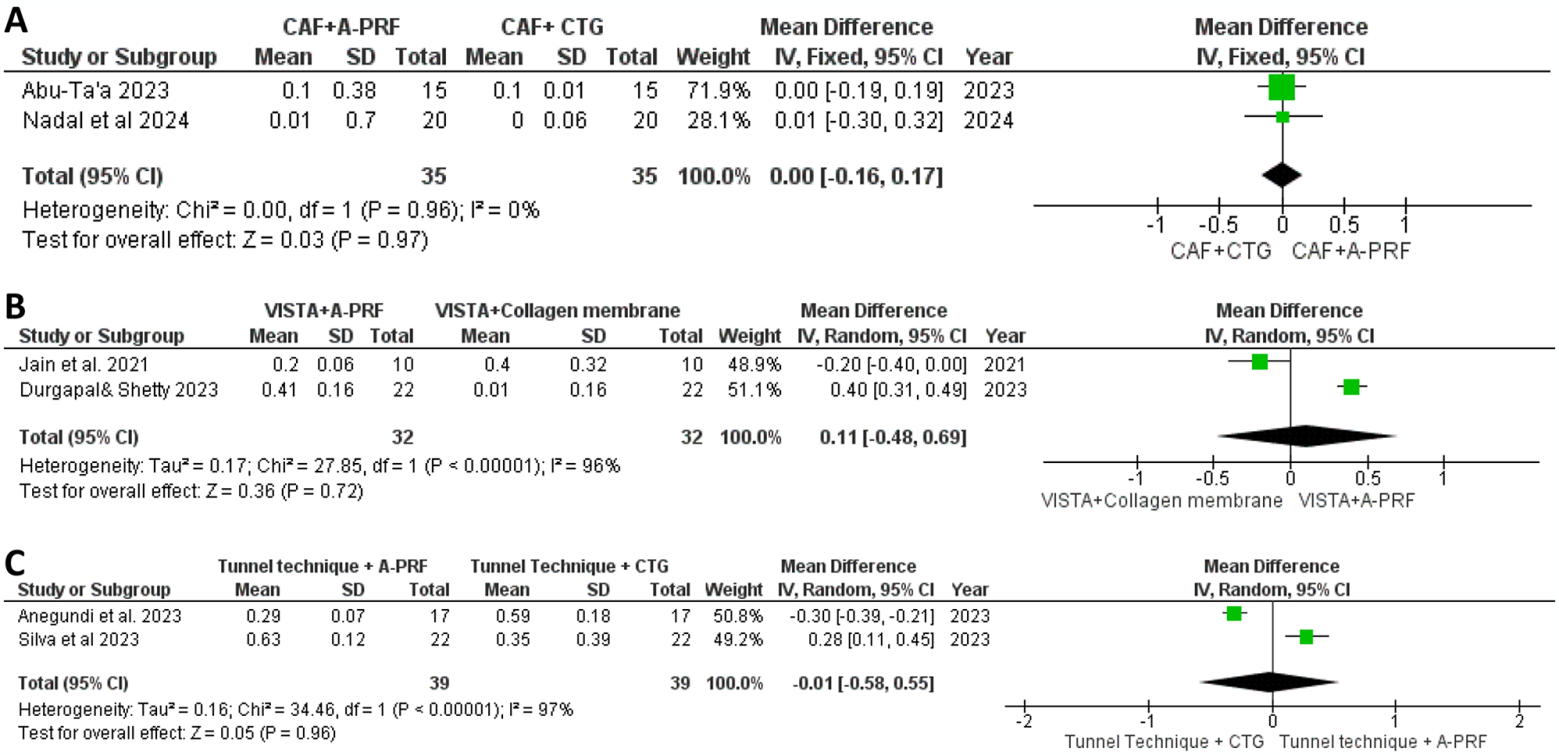
Forest plot for the PPD reduction after 6 months. **A**: Comparison between CAF + A-PRF vs. CAF + CTG. **B**: Comparison between VISTA + A-PRF vs. VISTA + collagen membrane. **C**: Comparison between tunnel technique + A-PRF vs. tunnel technique + CTG

In Fig. 6A, our meta-analysis investigated PPD in two studies comparing CAF with A-PRF to CAF with CTG, employing a fixed-effects model. Importantly, the analysis showed no heterogeneity (I² = 0%), indicating consistent effects across the studies [35, 36]. Figure 6B presents our analysis of PPD outcomes from two studies comparing VISTA with A-PRF to VISTA with a collagen membrane. Using a random-effects model, we found a mean difference of 0.11, with substantial heterogeneity (I² = 96%) and no statistically significant difference between groups (*P* = 0.72), suggesting variability in outcomes without a clear treatment advantage in reducing PPD [31, 32]. Figure 6C illustrates our meta-analysis comparing the tunnel technique with A-PRF to the tunnel technique with CTG, showing a mean difference of 0.01 in PPD. Significant heterogeneity (I² = 97%) was observed, and the difference between groups was not statistically significant (*P* = 0.96), indicating no clear superiority in reducing PPD between the treatments at the six-month [33, 34]. 

#### Clinical attachment level

**Fig. 7:**
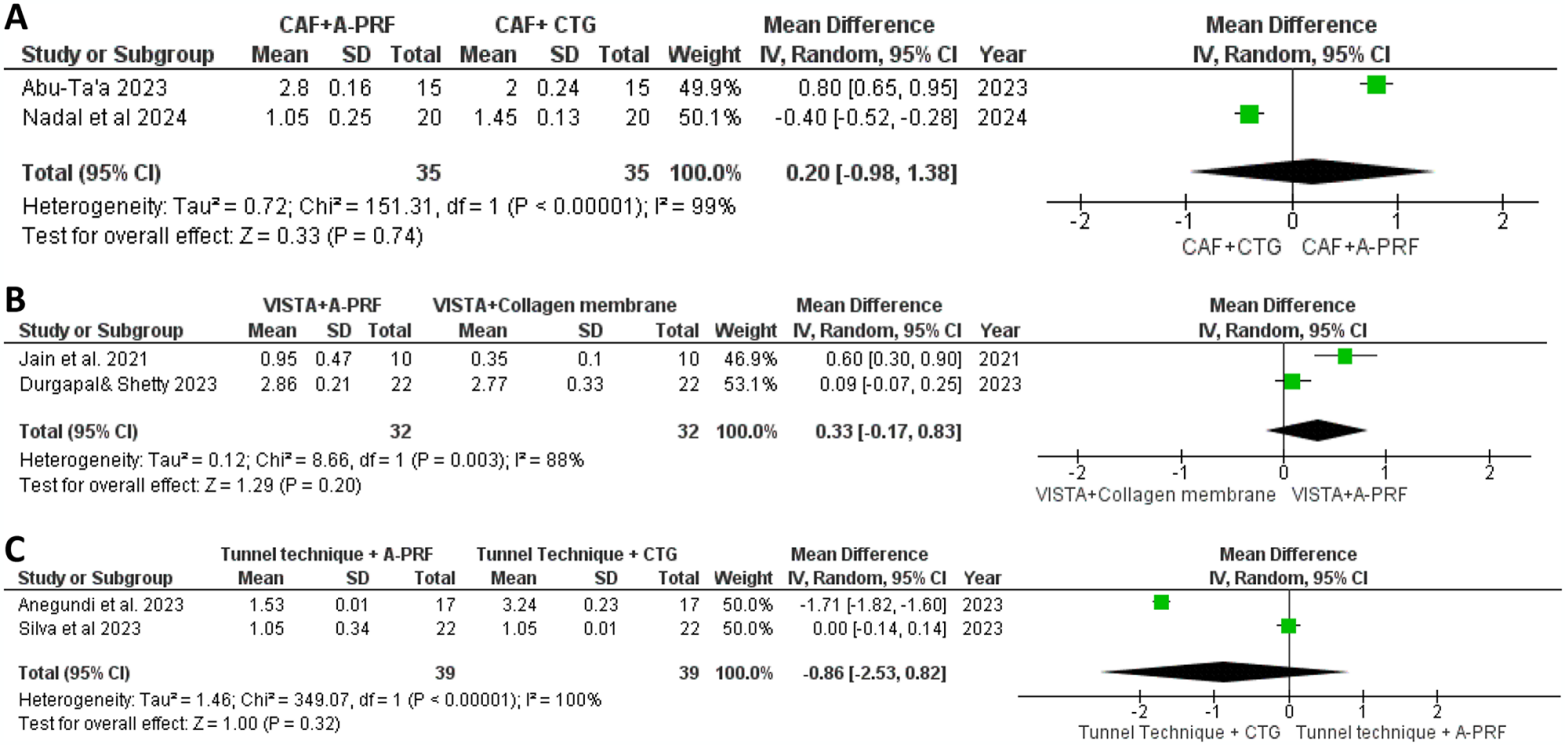
Forest plot for the CAL change after 6 months. **A**: Comparison between CAF + A-PRF vs. CAF + CTG. **B**: Comparison between VISTA + A-PRF vs. VISTA + collagen membrane. **C**: Comparison between tunnel technique + A-PRF vs. tunnel technique + CTG

In Fig. 7A, our meta-analysis compared CAL between CAF with A-PRF and CAF with CTG in two studies, employing a random-effects model that revealed a mean difference of 0.20. Despite significant heterogeneity (I²=99%), no statistically significant difference in CAL was found between the treatment groups (*P* = 0.74) [35, 36]. Figure 7B focused on CAL outcomes from studies comparing VISTA with A-PRF to VISTA with a collagen membrane, showing a mean difference of 0.33 with significant heterogeneity (I²=88%). Similarly, no significant difference in CAL was observed between the groups (*P* = 0.20) [31, 32]. Figure 7C illustrated our meta-analysis comparing the tunnel technique with A-PRF to the tunnel technique with CTG, revealing a mean difference of 0.86 in CAL. Despite high heterogeneity (I²=100%), the analysis showed no statistically significant difference (*P* = 0.32), indicating no clear effect in improving CAL between the treatments at the six-month mark [33, 34]. 

## Discussion

A-PRF is a recently developed variation of PRF that presents specific biological properties. It has not been systematically evaluated in the field of gingival recession treatment. To the best of our knowledge, this review is the first to assess the efficacy of A-PRF in treating gingival recession. We noticed that the research in which A-PRF was utilized for covering the gingival recession is still scarce. Consequently, our search strategy didn’t limit the date of publication. We aimed to include all the available research studies and to attain a higher heterogeneity among the results of included studies. We carefully selected our articles while adhering to the specific methodological protocols to achieve greater consistency in our results and avoid potential bias.

Our findings were based on 10 selected RCTs involving 146 participants and 457 recession defects. We focused on the clinical outcomes concerning the utilization of A-PRF in treating different classes of gingival recession, to compare its efficacy with other treatment methods. This methodology may help the clinicians to take an informed decision regarding its use in treating gingival recessions.

Our analysis included a variety of surgical techniques and intervention groups to explore the effect of A-PRF across the studies. The effectiveness of A-PRF in combination with various procedures such as VISTA, CAF, tunneling techniques, and the pinhole surgical technique was evaluated. Two studies investigated the outcomes of VISTA combined with A-PRF versus VISTA with a collagen membrane. A-PRF was compared to CAF alone in another 2 studies. Additionally, tunnel technique combined with A-PRF was compared to the same technique combined with CTG. Pinhole surgical technique with A-PRF versus pinhole surgical technique were compared. In addition, 2 studies measured the effect of CAF with A-PRF versus CAF combined with CTG. Lastly, the final study investigated the effect of CAF combined with A-PRF versus CAF combined with L-PRF.

There are no specific guidelines concerning the optimal application of A-PRF for treating gingival recession. This includes the ideal thickness of A-PRF, the number of A-PRF membranes required per treatment site, and the most effective surgical techniques for utilizing A-PRF.

We found slight variations in the centrifugation speed and time among the included studies. While A-PRF preparation follows a specific centrifugation protocol (decrease centrifugation speed and longer period), mild variations exist throughout studies due to variations in equipment, processing time, and clinical application. These minor deviations are regularly found in clinical research as protocols evolve or are adapted based totally on practitioner preference or the design of the studies [17, 25, 26, 31, 34, 36]. In our overview, we aimed to provide a complete review of the available literature, even though slight variations in A-PRF preparation times and speeds have been defined. However, we did not intend to show that A-PRF itself represents more than one protocol but alternatively recognized the variability determined in its application across different study types.

The variation in centrifugation speed and duration of A-PRF may affect the amount of growth factors and the healing of tissue potentially leading to variation of its clinical efficiency. So, future studies should focus on standardizing A-PRF preparation methods to enhance reproducibility and comparability across studies.

The follow-up periods of post treatment assessment differed among the included studies. Most of the studies conducted follow-up at 3 and 6 months. Some studies extended their follow-ups to 6 and 12 months aiming to conduct long-term evaluation of the outcomes.

We conducted a meta-analysis of the primary and secondary outcomes across the various treatment comparisons for gingival recession. Our meta-analysis of the RD revealed that a significant reduction in RD favoring the CTG group in comparison of CAF with A-PRF to CAF with CTG. This shows that CTG may provide superior outcomes in covering gingival recession when compared to A-PRF. CTG provides a stable base for gingival tissue formation as well as epithelial attachment [38–40]. However, A-PRF membrane may provide this structural support initially due to its reliance on the body’s own processes to organize into a functional matrix [41, 42]. 

Moreover, our analysis revealed a significant difference in RD favoring VISTA with A-PRF in comparison to VISTA with a collagen membrane. It may be attributed to the biological properties and regenerative effects of A-PRF [41, 42]. While collagen membrane showed improvement of the periodontal outcomes after surgery, it has comparatively less regenerative ability than A-PRF. It serves as a barrier to guide tissue regeneration; however, it lacks the cellular components and the growth factors available in A-PRF. Despite the limited number of studies included in our review, the low regenerative ability of collagen membrane explains the less favorable outcomes observed in our analysis [43–45]. 

We observed no significant difference in RW among all the studies included in the meta-analysis. The limited number if available studies, variability in patients, surgical procedures and the follow-up durations may result in high heterogeneity among the studies with diluting the overall effect, making it challenging to detect significant differences in RW. Consequently, further research is required to explore the possible potential factors causing the observed heterogeneity and validate these results across different populations.

Our meta-analysis revealed no statistically significant differences in WKG, PPD, or CAL between A-PRF and CTG across various surgical techniques, despite substantial heterogeneity. This lack of statistical difference makes A-PRF a comparative alternative to CTG for soft tissue regeneration in the treatment of gingival recession. The comparable effect of A-PRF may be due to its regenerative potential [41, 42]. A-PRF can enhance leukocyte degranulation and cytokine release, transitioning from proinflammatory mediators to anti-inflammatory cytokines as well as growth factors. These components play a significant role in various healing processes. A-PRF, based on the low-speed centrifugation concept, enables a greater number of leukocytes to be enclosed within the fibrin matrix and continuous releasing of growth factors for 14 days [13, 41, 42, 46]. 

We found a high heterogeneity among studies. The detected heterogeneity among studies may be due to the variability in the surgical techniques, design of the study, the measured outcomes, the protocols of the study, and other patient-related factors as genetic factors, oral hygiene, and periodontal health. Hence, we suggest future well-designed studies including more standardized methods of evaluation of the gingival recession outcomes.

While CTG has shown a higher effectiveness in treating gingival recession but come with several limitations. These include the morbidity of the donor site, the potential surgical complications and the increased patient discomfort. Moreover, more surgical time and technical skills with limited tissue availability may decrease the utilization of CTG in root coverage procedures. Additionally, tissue mismatches may result in less satisfactory esthetic outcomes. All these challenges necessitate the consideration of alternative treatments as A-PRF. ^[41]^

## Conclusion

We measured the effect of A-PRF in treating gingival recession in 10 RCTs. Our analysis showed that the combination of A-PRF with various surgical techniques, such as VISTA, tunneling, and pinhole surgical techniques, demonstrated promising outcomes but varied by comparison group. We observed that CTG with CAF showed a higher reduction in RD in comparison to A-PRF with CAF. This review indicated no statistical or clinical differences in RW, WKG, PPD, and CAL between the study and control groups. This lack of statistical difference makes A-PRF a comparative alternative to CTG or collagen membrane. However, further clinical trials with more participants are required to strengthen the analysis of the clinical outcomes.

A-PRF can improve treatment of gingival recession by enhancing tissue regeneration and decreasing the need for greater invasive methods. It is especially beneficial when mixed with surgical techniques such as CAF, as it accelerates healing and helps new tissue formation. Since it uses the patient’s own blood, there is no need for extra grafting, making the technique less painful. A-PRF also can be utilized in dental surgical procedures, now not just for gingival recession, but also for repairing bone and tissue around dental implants.

### Limitations

While the current systematic review provides significant information about the first meta-analysis to evaluate the effectiveness of A-PRF in treating gingival recession, it is essential to address certain limitations. We found some variations in the preparation time and speed among the included clinical trials that may cause inconsistent effects. Additionally, whilst it releases growth factors, this occurs only for a short period, which might not be sufficient for large surgical defects. In addition, the low number of available trials limits the generalization of data. Hence, we recommend further longitudinal multi-center trials including a larger size population to evaluate the outcomes of surgical treatment of gingival recession. Moreover, we found high heterogeneity among studies in terms of surgical techniques, study designs, sample size, and follow-up duration that may have a significant effect in the outcomes of our analysis.

## Data Availability

The data that support the findings of this study are available from the corresponding author upon reasonable request.
